# 2023年第3版《NCCN肿瘤临床实践指南：非小细胞肺癌》更新解读

**DOI:** 10.3779/j.issn.1009-3419.2023.102.18

**Published:** 2023-06-20

**Authors:** Lingling ZHU, Ting WANG, Juan WU, Xiaoqian ZHAI, Qiang WU, Hanyu DENG, Changlong QIN, Long TIAN, Qinghua ZHOU

**Affiliations:** ^1^610041 成都，四川大学华西医院肺癌中心; ^1^Lung Cancer Center, West China Hospital, Sichuan University, Chengdu 610041, Chin; ^2^610041 成都，四川大学华西医院门诊部; ^2^Outpatient Department, West China Hospital, Sichuan University, Chengdu 610041, China

**Keywords:** 临床实践, 肺肿瘤, NCCN指南, 指南解读, Clinical practice, Lung neoplasms, NCCN guideline, Guideline interpretation

## Abstract

肺癌是我国发病率和死亡率均居首位的恶性肿瘤，非小细胞肺癌（non-small cell lung cancer, NSCLC）是肺癌的主要病理学亚型。2023年4月13日，美国国立综合癌症网络（National Comprehensive Cancer Network, NCCN）发布了2023年第3版《NCCN肿瘤临床实践指南：非小细胞肺癌》，其反映了国际肺癌研究的最新进展。本文将对新版《指南》主要更新内容进行解读，并与2022年第3版NCCN指南相比较，旨在为我国临床医务人员诊治NSCLC提供参考。

## Abstract (en)

Lung cancer is the malignant tumor with the highest morbidity and mortality in China. Non-small cell lung cancer (NSCLC) is the main pathological subtype of lung cancer. On April 13, 2023, the National Comprehensive Cancer Network (NCCN) released the third edition of the 2023 NCCN Oncology Clinical Practice Guidelines: Non-small Cell Lung Cancer, which reflects the latest advances in international lung cancer research. This article will interpret the main updated contents of the new edition of the guidelines, and compare it with the third edition of the NCCN guidelines in 2022, so as to provide references about the diagnosis and treatment of NSCLC for clinical medical personnel in China.

美国国立综合癌症网络（National Comprehensive Cancer Network, NCCN）是由全美32家顶级癌症中心组成的非营利性组织，其所制定的《NCCN肿瘤临床实践指南》为指导全球临床医务人员的正确临床治疗决策做出了重要贡献。本文将重点关注、解读和比较最新发布的《NCCN肿瘤临床实践指南：非小细胞肺癌》（下文简称“《指南》”）与2022年第3版指南的更新内容及其差异。指南以临床研究数据为依据，确保肿瘤领域专家和患者均能获取最新的癌症预防、筛选和治疗方法，从而达到最佳疗效。在非小细胞肺癌（non-small cell lung cancer, NSCLC）治疗领域，2023年4月13日，NCCN发布了2023年第3版《指南》^[1]^。与2022年第3版^[2]^指南比较，2023年第3版《指南》更新主要集中在诊断评估原则、多发性肺癌诊治、免疫治疗、靶向治疗、围手术期系统治疗、术后放疗、分子和生物标志物检测等方面。因此，本文将对主要更新内容进行解读。

## 1 诊断评估

新版《指南》对术前活检建议变动较大，临床高度怀疑肺癌（基于危险因素和影像学表现）且术前无需活检的患者的临床分期从I期-II期更新为IA期，新增“临床分期为IB期或更晚分期的患者，术前活检有助于该部分患者接受术前全身治疗”。这一改动基于III期随机对照临床试验CheckMate 816的研究结果，该研究结果显示IB期或分期更晚NSCLC患者术前接受纳武利尤单抗（Nivolumab）联合化疗的新辅助治疗相较于新辅助化疗，能够显著延长无事件生存期（event-free survival, EFS），并提高病理完全缓解（pathological complete response, pCR）率^[3]^。术前支气管镜检查是组织诊断和/或纵隔分期[超声支气管镜（endobronchial ultrasound, EBUS）]的首选。对于<3 cm的周围型肺癌（肺外三分之一），当计算机断层扫描（computed tomography, CT）和正电子发射计算机断层显像（positron emission tomography/CT, PET/CT）检查纵隔淋巴结为阴性时，治疗前纵隔淋巴结病理学评估并非必须。中心型肿瘤则推荐行侵袭性纵隔分期，对于医学上认为不能手术的纵隔淋巴结转移患者，纵隔活检通常是首选。但在一些特殊的患者中，风险可能大于获益。中心型肺癌如需术前活检或改善气道梗阻，则推荐术前行EBUS。

对于多学科评估，旧版《指南》限定于“IIB期和III期”患者，而新版《指南》对于考虑一种以上治疗模式（手术、放疗或系统性治疗）的患者，均推荐进行多学科综合评估，并未根据分期区分适宜患者。同时建议EBUS作为病理诊断和/或纵隔淋巴结分期的方法，必要时EBUS可对2R/2L、3P、4R/4L、7、10R/10L、11组-13组淋巴结和其他肺门淋巴结进行活检，其中3P、11组-13组淋巴结为新版《指南》新增内容。

对于靶向治疗耐药的检测，新版《指南》提出新观点：对于任何在靶向治疗期间出现进展的患者，组织学转化（如小细胞）是潜在的耐药机制之一，应考虑对相关病灶进行组织活检，以评估形态学并分析生物标志物的变化。组织学转化和其他脱靶分子改变是奥希替尼（Osimertinib）的早期耐药常见机制，与不良预后有密切关系^[4]^。

## 2 治疗

### 2.1 多发性肺癌诊治

确定多发性肺癌是“单/多原发、多转移”还是“多原发（含同时性和异时性）”对于患者的后续治疗极其重要。目前虽已建立了多发性肺癌的诊断标准，但尚无标准治疗方案^[5]^。多发性肺癌多为转移瘤，但若病理检查提示为不同病理类型的病变（如鳞状细胞癌或腺癌），通常意味着病变为多原发性肺癌^[6]^，而相同病理类型的病变也有可能不是转移癌。值得注意的是，新版《指南》针对多发性肺癌首先推荐多学科诊疗（multi-disciplinary treatment, MDT）模式，同时精简了诊治和随访流程（图1）。对于疾病稳定或进展缓慢的结节可随访观察，而对于进展迅速的结节，如果病变呈加速生长或实性成分增加或氟代脱氧葡萄糖（fluorodeoxyglucose, FDG）摄取增加，即使病变很小，也应进行局部治疗。局部治疗应首选外科手术治疗，其次选择放疗或影像引导下热消融（image-guided thermal ablation, IGTA）；如患者不适合局部治疗，则应采取姑息性治疗或观察。对于多原发癌经姑息性化疗±局部姑息治疗或观察后进展的患者，可参照复发和转移的治疗方案或转移性疾病的全身治疗方案进行。

**图1:**
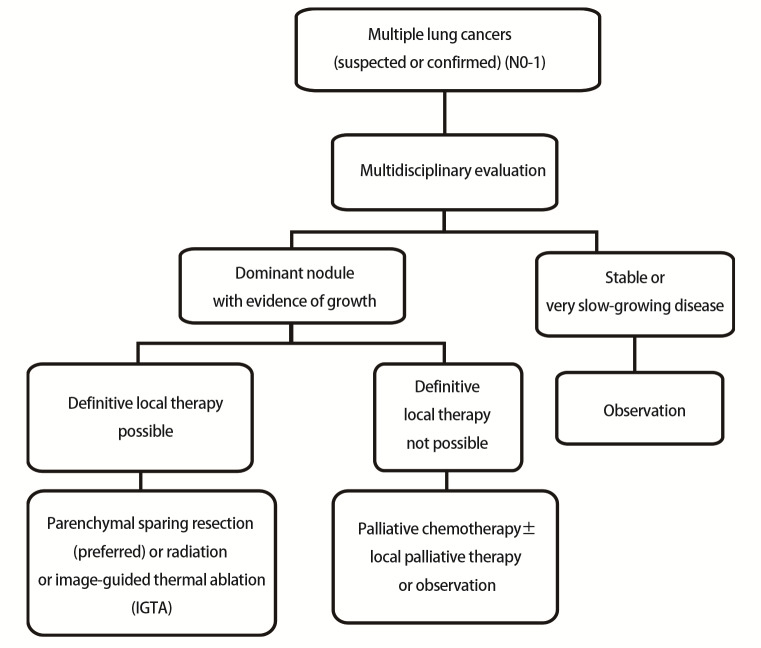
多发性肺癌诊治流程图

### 2.2 免疫治疗

新版《指南》将2022版指南新辅助和辅助治疗全身治疗方案部分修改为围手术期全身治疗方案，后者包括新辅助治疗方案、辅助治疗方案和既往辅助治疗后的全身治疗，使得新版《指南》中该部分思路更为明晰。

#### 2.2.1 新辅助治疗

针对新辅助治疗，新版《指南》推荐临床IB期-IIIB（T3, N2）期患者根据程序性细胞死亡配体1（programmed cell death ligand 1, PD-L1）表达、表皮生长因子受体（epidermal growth factor receptor, EGFR）突变和间变性淋巴瘤激酶（anaplastic lymphoma kinase, ALK）重排情况选择药物组合。CheckMate 816研究^[3]^结果显示术前新辅助治疗应用纳武利尤单抗联合化疗与单纯化疗相比，可显著延长EFS，并提高pCR率。在新辅助化疗基础上加用纳武利尤单抗未增加不良事件发生率，也未降低手术可行性。据此，对于肿瘤≥4 cm（分期至少为IB期）或淋巴结阳性（分期至少为IIB期）且无免疫检查点抑制剂（immune checkpoint inhibitors, ICIs）使用禁忌[包括活动性或既往有自身免疫性疾病和/或目前正在使用免疫抑制剂；某些已被证明与程序性细胞死亡受体1（programmed cell death 1, PD-1）/PD-L1抑制剂疗效较差相关的致癌驱动基因，如EGFR 19外显子缺失（EGFR 19del）或L858R突变、ALK重排表达阳性]，新版《指南》推荐应用纳武利尤单抗联合化疗的新辅助治疗（表1）。如患者不能使用ICIs，则推荐使用含铂双药治疗（表2）。

**表1:** 新辅助治疗方案

Neoadjuvant systemic therapy	Application plan of drugs
Platinum-doublet chemotherapy^a^	Carboplatin AUC 5 or AUC 6 day 1, Paclitaxel 175 mg/m² or 200 mg/m² day 1 (any histology); Cisplatin 75 mg/m² day 1, Pemetrexed 500 mg/m² day 1 (non-squamous histology); Cisplatin 75 mg/m² day 1, Gemcitabine 1,000 mg/m² or 1,250 mg/m² days 1 and 8 (squamous histology); Cisplatin 75 mg/m² day 1, Paclitaxel 175 mg/m² or 200 mg/m² day 1 (any histology)
Chemotherapy regimens for patients Not candidates for cisplatin-based therapy^b^	Carboplatin AUC 5 or AUC 6 day 1, Pemetrexed 500 mg/m² day 1 (non-squamous histology); Carboplatin AUC 5 or AUC 6 day 1, Gemcitabine 1,000 mg/m² or 1,250 mg/m² days 1 and 8 (squamous histology)
Preferred (non-squamous)	Cisplatin 75 mg/m^2^ day 1, Pemetrexed 500 mg/m^2^ day 1, every 21 days for 4 cycles
Preferred (squamous)	Cisplatin 75 mg/m^2^ day 1, Gemcitabine 1,250 mg/m^2^ days 1 and 8, every 21 days for 4 cycles; Cisplatin 75 mg/m^2^ day 1, Docetaxel 75 mg/m^2 ^day 1, every 21 days for 4 cycles
Other recommended	Cisplatin 50 mg/m^2 ^days 1 and 8; Vinorelbine 25 mg/m^2^ days 1, 8, 15 and 22, every 28 days for 4 cycles; Cisplatin 100 mg/m^2^ day 1, Vinorelbine 30 mg/m^2^ days 1, 8, 15 and 22, every 28 days for 4 cycles; Cisplatin 75 mg/m^2^-80 mg/m^2^ day 1, Vinorelbine 25 mg/m^2^-30 mg/m^2^ days 1 and 8, every 21 days for 4 cycles; Cisplatin 100 mg/m^2^ day 1, Etoposide 100 mg/m^2^ days 1-3, every 28 days for 4 cycles
Chemotherapy regimens for patients Not candidates for cisplatin-based therapy	Carboplatin AUC 6 day 1, Paclitaxel 200 mg/m^2^ day 1, every 21 days for 4 cycles; Carboplatin AUC 5 day 1, Gemcitabine 1,000 mg/m^2 ^days 1 and 8, every 21 days for 4 cycles (squamous histology); Carboplatin AUC 5 day 1, Pemetrexed 500 mg/m^2^ day 1, every 21 days for 4 cycles (non-squamous histology)

^a^: Neoadjuvant systemic therapy in patients candidates for immune checkpoint inhibitors; Nivolumab 360 mg and platinum-doublet chemotherapy every 3 weeks for 3 cycles; ^b^: Neoadjuvant systemic therapy for patients Not candidates for immune checkpoint inhibitors; All chemotherapy regimens listed can be used for sequential chemotherapy/radiotherapy. AUC: area under the curve.

**表2:** 辅助治疗方案

Adjuvant systemic therapy	Application plan of drugs
Preferred (non-squamous)	Cisplatin 75 mg/m^2^ day 1, Pemetrexed 500 mg/m^2^ day 1, every 21 days for 4 cycles
Preferred (squamous)	Cisplatin 75 mg/m^2^ day 1, Gemcitabine 1,250 mg/m^2^ days 1 and 8, every 21 days for 4 cycles; Cisplatin 75 mg/m^2^ day 1, Docetaxel 75 mg/m^2^ day 1, every 21 days for 4 cycles
Other recommended	Cisplatin 50 mg/m^2^ days 1 and 8; Vinorelbine 25 mg/m^2^ days 1, 8, 15 and 22, every 28 days for 4 cycles; Cisplatin 100 mg/m^2^ day 1, Vinorelbine 30 mg/m^2^ days 1, 8, 15 and 22, every 28 days for 4 cycles; Cisplatin 75 mg/m^2^-80 mg/m^2^ day 1, Vinorelbine 25 mg/m^2^-30 mg/m^2^ days 1 and 8, every 21 days for 4 cycles; Cisplatin 100 mg/m^2^ day 1, Etoposide 100 mg/m^2^ days 1-3, every 28 days for 4 cycles
Chemotherapy regimens for patients Not candidates for Cisplatin-based therapy	Carboplatin AUC 6 day 1, Paclitaxel 200 mg/m^2^ day 1, every 21 days for 4 cycles; Carboplatin AUC 5 day 1, Gemcitabine 1,000 mg/m^2^ days 1 and 8, every 21 days for 4 cycles; Carboplatin AUC 5 day 1, Pemetrexed 500 mg/m^2^ day 1, every 21 days for 4 cycles (non-squamous histology)

All chemotherapy regimens listed above can be used for sequential chemotherapy/radiotherapy.

#### 2.2.2 辅助治疗

新版《指南》关于免疫辅助治疗的部分更新，主要集中在两个方面。首先，基于AEGEAN研究^[7]^结果，删除了“不推荐度伐利尤单抗（Durvalumab）用于术后辅助免疫治疗的内容”。AEGEAN研究^[7]^是一项随机、双盲、安慰剂对照、全球多中心的III期临床研究，旨在评估可切除的II期-III期NSCLC患者术前新辅助度伐利尤单抗加化疗、术后接受度伐利尤单抗单药治疗的疗效和安全性。结果显示，在可切除的NSCLC患者中，术前新辅助度伐利尤单抗联合化疗+辅助度伐利尤单抗单药与安慰剂对照相比，EFS分别为未达到（not reached, NR） vs 25.9个月（HR=0.68, P=0.0039），pCR率分别为17.2%和4.3%，在pCR方面有显著改善。

其次，基于Impower 010研究^[8]^结果，美国食品药品监督管理局（Food and Drug Administration, FDA）已批准PD-L1抑制剂阿替利珠单抗（Atezolizumab）在手术和含铂化疗之后辅助治疗PD-L1≥1%的II期-IIIA期NSCLC患者。对于肺上沟瘤（T3浸润，N0-1）、肺上沟瘤（T4扩散，N0-1）或胸壁、接近气道或纵隔（T3浸润，N0-1；可切除T4扩散，N0-1）患者术后辅助治疗，新版《指南》推荐阿替利珠单抗适用于EGFR 19del/L858R突变或ALK重排阴性、PD-L1≥1%、辅助化疗后患者。对于分散的肺结节，同一肺叶（T3, N0-1），或同侧非原发肺叶（T4, N0-1）术后患者，新版《指南》推荐化疗随后给予阿替利珠单抗或帕博利珠单抗（Pembrolizumab）或奥希替尼。根治性手术是I期-II期和部分IIIA期NSCLC的首选治疗方法。PEARLS/KEYNOTE-091（NCT02504372）^[9]^是一项随机对照、三盲III期临床试验，旨在评估帕博利珠单抗对比安慰剂作为IB（≥4 cm）期、II期和IIIA期NSCLC患者根治术后辅助治疗的有效性和安全性。研究结果显示，帕博利珠单抗显著改善中位无病生存期（disease-free survival, DFS）（53.6个月 vs 42.0个月；HR=0.76；95%CI: 0.63-0.91；P=0.0014）。据此，新版《指南》新增帕博利珠单抗对于II期、IIIA期、IIIB（T3, N2）期、肺上沟瘤（T3浸润，N0-1）、肺上沟瘤（T4扩散，N0-1）、胸壁、接近气道或纵隔（T3浸润，N0-1；可切除T4扩散，N0-1）根治性切除术后患者的术后辅助免疫治疗，适用于EGFR 19del/L858R突变或ALK重排阴性且既往接受过辅助化疗的患者。值得注意的是，新版《指南》指出，针对PD-L1<1%的患者，免疫治疗是否能使其获益尚不清楚。

#### 2.2.3 既往辅助治疗后的全身治疗

新版《指南》新增了既往辅助系统治疗后的全身治疗内容，主要集中在三个方面：（1）奥希替尼用于完全切除的IB期-IIIA期和IIIB（T3, N2）期EGFR突变阳性（19del/L858R）NSCLC患者，这些患者既往接受过辅助化疗或不适合铂类化疗；（2）阿替利珠单抗用于既往接受辅助化疗且完全切除的“IB期-IIIA期和IIIB（T3, N2）期”或高危IIA期PD-L1≥1%的NSCLC患者最多1年，并且无ICIs使用禁忌；（3）帕博利珠单抗用于既往接受辅助化疗且完全切除的“IIB-IIIA期、IIIB期（T3, N2），或高危IIA期”EGFR 19del/L858R突变或ALK重排阴性的患者，并且无ICIs使用禁忌（表3）。

**表3:** 既往辅助系统治疗后的全身治疗方案

Agents	Target population
Osimertinib 80 mg daily	Osimertinib for patients with completely resected stage IB-IIIA or stage IIIB (T3, N2) NSCLC and positive for EGFR mutations (exon 19 deletion, exon 21 L858R mutations) who received previous adjuvant chemotherapy or are ineligible to receive platinum-based chemotherapy
Atezolizumab 840 mg every 2 weeks, 1,200 mg every 3 weeks, or 1,680 mg every 4 weeks for up to 1 year	Atezolizumab for patients with completely resected stage IIB-IIIA, stage IIIB (T3, N2), or high-risk stage IIA NSCLC with PD-L1≥1% and negative for EGFR exon 19 deletion or exon 21 L858R mutations or ALK rearrangements who received previous adjuvant chemotherapy and with no contraindications to immune checkpoint inhibitors
Pembrolizumab 200 mg every 3 weeks or 400 mg every 6 weeks for up to 1 year	Pembrolizumab for patients with completely resected stage IIB-IIIA, stage IIIB (T3, N2), or high-risk stage IIA NSCLC and negative for EGFR exon 19 deletion or exon 21 L858R mutations or ALK rearrangements who received previous adjuvant chemotherapy and with no contraindications to immune checkpoint inhibitors

NSCLC: non-small cell lung cancer; EGFR: epidermal growth factor receptor; PD-L1: programmed cell death ligand 1; ALK: anaplastic lymphoma kinase.

#### 2.2.4 晚期肺癌的免疫治疗

新版《指南》针对晚期肺癌患者提出了双免疫联合治疗，即在PD-L1抑制剂的基础上，加用人细胞毒性T淋巴细胞抗原4（cytotoxic T-lymphocyte antigen 4, CTLA-4）单抗。新版《指南》推荐的两种CTLA-4单抗分别为伊匹木单抗（Ipilimumab）和替西木单抗（Tremelimumab）。伊匹木单抗可与纳武利尤单抗联用（1 类推荐），或联用基础上增加2个周期化疗（1类推荐）。纳武利尤单抗+伊匹木单抗+2个周期化疗与单独化疗相比显著延长了患者的总生存期（overall survival, OS），并且具有良好的风险-获益特征^[10]^。与化疗相比，在度伐利尤单抗联合化疗的基础上加用有限疗程的替西木单抗（1类推荐）显著改善了患者的OS和无进展生存期（progression-free survival, PFS），且未增加耐受性负担^[11]^。

新版《指南》更新了PD-L1≥50%和1%-49%晚期NSCLC患者的一线治疗和维持治疗（表4，表5）。KEYNOTE-042（NCT02220894）研究^[12]^在PD-L1≥1%的进展期/转移性NSCLC一线治疗中比较帕博利珠单抗与含铂化疗方案的疗效与安全性的开放性III期研究，结果显示帕博利珠单抗组相比化疗组明显改善患者生存，而且毒性更低。

**表4:** PD-L1≥50%晚期转移性疾病的免疫治疗方案

Pathological subtype	First-line therapy		Continuation maintenance
	Preferred	Other recommended	
Adenocarcinoma, large cell, NSCLC not otherwise specified	Pembrolizumab (category 1); Carboplatin or Cisplatin+ Pemetrexed+Pembrolizumab (category 1); Atezolizumab (category 1); Cemiplimab-rwlc (category 1)	Carboplatin+Paclitaxel+ Bevacizumab+Atezolizumab (category 1); Carboplatin+albumin-bound Paclitaxel+Atezolizumab; Nivolumab+Ipilimumab+Pemetrexed+ Carboplatin or Cisplatin (category 1); Cemiplimab-rwlc+Paclitaxel+Carboplatin or Cisplatin (category 1); Cemiplimab-rwlc+Pemetrexed+Carboplatin or Cisplatin (category 1); Tremelimumab-actl+Durvalumab+Carboplatin+albumin-bound Paclitaxel (category 2B); Tremelimumab-actl+Durvalumab+Carboplatin or Cisplatin+Pemetrexed (category 2B)	Pembrolizumab (category 1); Pembrolizumab+Pemetrexed (category 1); Atezolizumab+Bevacizumab (category 1); Atezolizumab; Nivolumab+Ipilimumab (category 1); Cemiplimab-rwlc (category 1); Cemiplimab-rwlc±Pemetrexed (category 1); Durvalumab±Pemetrexed
Squamous cell carcinoma	Pembrolizumab (category 1); Carboplatin+Paclitaxel or albumin-bound Paclitaxel+ Pembrolizumab (category 1); Atezolizumab (category 1); Cemiplimab-rwlc (category 1)	Nivolumab+Ipilimumab+Paclitaxel+Carboplatin (category 1); Cemiplimab-rwlc+Paclitaxel+Carboplatin or Cisplatin (category 1); Tremelimumab-actl+ Durvalumab+Carboplatin+albumin-bound Paclitaxel (category 2B); Tremelimumab-actl+Durvalumab+Carboplatin or Cisplatin+Gemcitabine (category 2B)	Pembrolizumab (category 1); Atezolizumab; Nivolumab+Ipilimumab (category 1); Cemiplimab-rwlc (category 1); Durvalumab

**表5:** PD-L1 1%-49%晚期或转移性NSCLC的免疫治疗方案

Pathological subtype	First-line therapy		Continuation maintenance
	Preferred	Other recommended	
Adenocarcinoma, large cell, NSCLC not otherwise specified	Carboplatin or Cisplatin+ Pemetrexed+ Pembrolizumab (category 1)	Carboplatin+Paclitaxel+Bevacizumab+Atezolizumab (category 1); Carboplatin+albumin-bound Paclitaxel+Atezolizumab; Nivolumab+Ipilimumab+Pemetrexed+Carboplatin or Cisplatin (category 1); Nivolumab+Ipilimumab (category 1); Cemiplimab-rwlc+Paclitaxel+Carboplatin or Cisplatin (category 1); Cemiplimab-rwlc+Pemetrexed+Carboplatin or Cisplatin (category 1); Tremelimumab-actl+Durvalumab+ Carboplatin+albumin-bound Paclitaxel (category 1); Tremelimumab-actl+Durvalumab+Carboplatin or Cisplatin+Pemetrexed (category 1)	Pembrolizumab (category 2B); Pembrolizumab+Pemetrexed (category 1); Atezolizumab+Bevacizumab (category 1); Atezolizumab; Nivolumab+Ipilimumab (category 1); Cemiplimab-rwlc±Pemetrexed (category 1); Durvalumab±Pemetrexed
Squamous cell carcinoma	Carboplatin+Paclitaxel or albumin-bound Paclitaxel+ Pembrolizumab (category 1)	Nivolumab+Ipilimuab+Paclitaxel+Carboplatin (category 1); Nivolumab+Ipilimumab (category 1); Cemiplimab-rwlc+Paclitaxel+Carboplatin or Cisplatin (category 1); Tremelimumab-actl+Durvalumab+ Carboplatin+albumin-bound Paclitaxel; Tremelimumab-actl+Durvalumab+Carboplatin or Cisplatin+Gemcitabine	Pembrolizumab; Nivolumab+Ipilimumab (category 1); Cemiplimab-rwlc (category 1); Durvalumab

### 2.3 靶向治疗

新版《指南》关于靶向治疗的更新内容主要集中在EGFR突变、KRAS突变和ROS1重排的药物治疗方面。

对于EGFR 20外显子插入突变（EGFR ex20ins）的后线治疗，新版《指南》推荐：如果患者既往接受过莫博替尼（Mobocertinib, TAK-788）治疗但未接受过埃万妥单抗（Amivantamab-vmjw, JNJ-61186372），则可以使用埃万妥单抗，反之亦然，因为两药具有不同的作用机制。EGFR ex20ins突变占EGFR所有突变的4%-12%。EGFR ex20ins突变对现有的第三代EGFR-酪氨酸激酶抑制剂（EGFR-tyrosine kinase inhibitors, EGFR-TKIs）敏感性均较差，响应率低（0%-9%）^[13]^ 。莫博替尼是一种原研、有效、口服、不可逆的小分子EGFR-TKIs，对EGFR ex20ins突变具有特殊活性^[14]^，而埃万妥单抗是一种具有免疫细胞导向活性的全人源EGFR-MET双特异性抗体。NCT02716116临床研究结果^[14,15]^显示莫博替尼在既往接受过治疗的EGFR ex20ins突变NSCLC患者中具有一定疗效，各亚组之间客观缓解率（objective response rate, ORR）无显著差异，并且安全性可控（ORR为28%，中位PFS为7.3个月，中位OS为24个月）；与传统标准治疗[二线多西他赛，ORR为14%，缓解持续时间（duration of response, DoR）为5.6个月-6.2个月]和免疫治疗相比（ORR为0%-25%，DoR未报道）疗效更好^[14,15]^。I期研究CHRYSALIS（NCT02609776）结果^[16]^显示，埃万妥单抗后线治疗ORR高达40%，中位PFS为8.3个月（95%CI: 6.5-10.9），中位OS为22.8个月（95%CI: 14.6-NR）。基于此，FDA已批准埃万妥单抗和莫博替尼用于EGFR ex20ins突变型NSCLC患者的后线治疗，但目前尚无临床数据提示两种药物序贯使用的合理顺序及疗效^[17]^ 。

KRAS突变是NSCLC一个重要的驱动基因，在西方人群中KRAS突变发生率达到20%-30%，在亚洲人群也达到7%-10%。中国肺腺癌中KRAS突变比例为8.3%，优势人群为男性、有吸烟史、病理为浸润性黏液型腺癌和实体型腺癌。KRAS突变位点中G12C最常见（33.6%），其次是G12D（23.9%）和G12V（22.1%）。II期研究KRYSTAL-1^[18]^评估了阿达格拉西布（Adagrasib）用于至少接受过一种含铂化疗和/或免疫治疗局部晚期或转移性KRAS^G12C^突变NSCLC患者的疗效和安全性。在112例基线有可测量病变的患者中，ORR为42.9%，疾病控制率（disease control rate, DCR）为79.5%，PFS为6.5个月，OS为12.6个月。如果既往没有使用过KRAS^G12C^靶向治疗，在一线治疗（或二线及以上）后，新版《指南》新增了KRAS^G12C^突变靶向药物阿达格拉西布，因此可以使用索托拉西布（Sotorasib）或阿达格拉西布。阿达格拉西布与索托拉西布作用机制相似，不建议在疾病进展时换药，但阿达格拉西布不良事件发生率较高^[19]^。另外，新版《指南》认为KRAS突变与EGFR-TKIs治疗反应性降低无相关性。

ROS1属于胰岛素受体家族的一种单体型受体酪氨酸激酶，多见于年轻、不吸烟或轻度吸烟肺腺癌患者，ROS1重排在NSCLC中发生率为1%-2%^[20]^。对ROS1重排治疗方面，新版《指南》推荐：如果之前未使用过，在使用一线药物色瑞替尼（Ceritinib）、克唑替尼（Crizotinib）或恩曲替尼（Entrectinib）出现脑转移症状后可考虑使用洛拉替尼（Lorlatinib）。洛拉替尼是一种口服的第三代ALK和ROS1抑制剂，具有良好的中枢神经系统渗透性^[21,22]^。在一项开放标签、单臂I期-II期试验（NCT01970865）^[21]^中，洛拉替尼在中枢神经系统转移性和克唑替尼耐药的ROS1阳性NSCLC患者中显示出较好的抗肿瘤活性，颅内肿瘤反应率达到64%（7/11）。

新版《指南》新增推荐曲妥珠单抗-德鲁替康（Trastuzumab Deruxtecan, DS-8201）或曲妥珠单抗-美坦新偶联物（Trastuzumab emtansine, T-DMI）用于全身治疗后进展的HER2阳性晚期或转移性肺腺癌/鳞癌患者。同时，新版《指南》新增晚期或转移性腺癌、大细胞癌、不明确型、鳞状细胞癌分子检测需包括ERBB2（HER2）。大约3%的非鳞NSCLC具有HER2基因突变，多见于女性、不吸烟的患者，且预后较差，脑转移发生率较其他突变更高。曲妥珠单抗-德鲁替康是一种抗体偶联药物（antibody-drug conjugate, ADC）^[23]^，该抑制剂可阻止癌细胞复制DNA，从而导致癌细胞死亡^[24]^。DESTINY-Lung01研究^[23]^是一项全球多中心、开放标签、双队列、II期临床研究，旨在评估曲妥珠单抗-德鲁替康治疗标准治疗耐药后HER2过表达或突变的转移性NSCLC患者的有效性和安全性。91例患者接受曲妥珠单抗-德鲁替康6.4 mg/kg，每三周一次，ORR为55%，中位DoR为9.3个月，中位PFS为8.2个月，中位OS为17.8个月。

### 2.4 术后放疗

新版《指南》对于局部晚期NSCLC的术后放疗进行了部分更新。对于通过手术后病理分期升至N2+且完全切除的临床I期/II期患者，LungART^[25]^和PORT-C^[26]^研究结果表明，术后放疗（postoperative radiation therapy, PORT）可显著改善局部控制率，但并未改善OS。因此，PORT可考虑用于特定的高危N2患者，如：包膜外侵犯、多站淋巴结受累、淋巴结清扫/采样不足、拒绝淋巴结采样或不耐受辅助全身治疗。为减少潜在的肺和心脏毒性，首选高适性放疗技术，如调强放疗（intensity modulated radiation therapy, IMRT）或质子治疗。

## 3 分子和生物标志物检测

对用于NSCLC分子诊断的组织标本采集，新版《指南》新增当组织极少时，实验室应采取相关技术最大限度地获得用于分子和辅助检测的组织样本，包括专门的小活检组织学流程以及用于诊断和预测性检测的“预”切片。

对于标本选择，新版《指南》新增：（1）使用组织和外周血的检测；（2）使用组织样本进行检测需要采集合适的样本；（3）使用外周血检测[最常见的是基于血浆的细胞游离DNA（circulating free DNA, cfDNA）和循环肿瘤DNA（circulating tumor DNA, ctDNA）检测）]可以与基于组织的检测联合，以实现推荐生物标志物的基因分型。对于血浆cfDNA/ctDNA检测，新版《指南》新增：在初始诊断时，如果及时进行基因组织检测的可行性不确定，同时进行ctDNA检测可能有助于生物标志物评估，以帮助临床医生进行治疗选择。血浆ctDNA是一种可在转移性NSCLC中识别ALK、BRAF、EGFR、HER2、RET、ROS1、MET 14外显子跳跃突变和其他致癌生物标志物的微创检查，血浆ctDNA检测被推荐在临床上广泛使用^[27⇓⇓-30]^。

## 4 小结

肺癌是在我国发生率和死亡率均居首位、对人群健康和生命威胁最大的肿瘤。新版《指南》对诊断评估原则、免疫治疗、靶向治疗及围手术期系统治疗改动较大。精简多发性肺癌的诊治流程，更有助于临床医生做出最佳医疗决策。而双免疫药物联合使用、术后放疗和分子标志物检测提供了新的治疗方法和见解。本文对新版《指南》主要更新内容进行解读，并与2022年第3版NCCN指南相比较，为我国临床医务人员诊治NSCLC提供了参考。

**Competing interests**

The authors declare that they have no competing interests.
